# Introduction—trade unions in times of austerity and development

**DOI:** 10.1007/s10624-020-09586-2

**Published:** 2020-03-04

**Authors:** Thomas McNamara, Manos Spyridakis

**Affiliations:** 1 La Trobe University, Melbourne, Australia; 2 Department of Social and Educational Policy, University of the Peloponnese, Corinth, Greece

This special issue seeks to understand how unions resist and are reshaped as they confront global capital and neoliberal austerity. It was born out of panels at the annual conferences of European Association of Social Anthropologists and the American Anthropology Association and through ongoing conversations about the similarities and differences between the challenges unions face from austerity in the Global North and from Global South aspirations for national ‘development’. The papers explore unions’ interactions with ‘actually existing neoliberalisms’, where the state and other actors express extreme faith in markets as policy devices, and in the superior efficiency of the private sector (Rizzo 2017). We describe unions responding to the growing criticism (from the right) that they imped the economic growth that would assist all of society and (from the left) that they represent a privileged and increasingly irrelevant minority of workers. While we explore unions’ attempts to dispute these narratives, our special issue is equally invested in detailing how unionism is re-constructed inside a community and, through this, how unions respond to, and co-create, legal, economic and cultural structures. In analysing how unionism shapes and responds to economic practices and workplace subjectivities, we foreground that the obligations and entitlements of being a ‘unionist’ and a ‘worker’ are negotiated through intra-community expectations and political histories. We therefore link unions to recent breakthroughs in the anthropological study of capitalism, where political economies are enacted through sociality and subjectivity (cf. Bear et al. 2015 and Hann and Parry 2018). Unionism is only beginning to be analysed using this framework, yet we show that unions are crucial to the concepts of self and relationships that shape and enable intra-national capitalisms (c.f. Kasmir 2014 and Lazar 2017). We explore how unions guide and respond to what it means to be a miner, steelworker or longshoreman, while simultaneously influencing what it means to be Zambian, Congolese, Greek or Argentinian. This enables us to provide new insights into the local configurations of labour, unionism and capitalism that are responding to unions’ global disempowerment. Our collection aspires to explore how unions are responding to the (perceived) growth of precarious work and to discourses presenting them as illegitimate or irrelevant; to detail how union practices and discourses connect the workplace to society; and to link ethnographies of unionism, grounded in a cultural anthropology tradition emphasizing ethos and discourse to their encompassing political economies, enabling an understanding of how unionism shapes specific capitalist projects. To guide our exploration, we utilize case studies from the North and South, where unions are differently challenged by, and differently respond to, global and local capitalisms.

## Austerity, ‘development’ and assaults on unionism

This special issue gathers papers on Argentina, Greece, Mexico, the Democratic Republic of the Congo and Zambia, nations among which unions’ political power varies greatly. Mexico and Argentina debatably have the strongest labour movements. However, the Argentinean Metalworkers Union has been deeply hurt by cuts to the steel industry and unions on Mexico’s border that are commonly government-controlled (Zlolniski 2019). In Zambia, nationalist politicians, international investors and global financial institutions have frequently posited a dichotomy between equitable wages and safe working conditions and the investment and tax dollars that signify development (Uzar 2017). In Greece, unions have had their powers reduced through debt-relief packages and are competing with fascist organizations for the hearts of disillusioned workers (Bithymitris 2011). While the challenges trade unions face differ between countries, key to many neoliberal socio-economic structures has been reducing the ability of unions to properly represent workers and the naturalization of increased employment precarity (Harvey 2007 and Standing 2011). While academic debates about actual historic rates of employment security are complex (Kasmir 2018), public discourse increasingly present insecure, temporary and low-waged work as inevitable, caused by a combination of mechanization and ‘the market’. A seemingly symbiotic relationship has developed between discourses of labour insecurity and narratives of union irrelevance. As work lives become increasingly fractured, commentators question the ability of unions to organize and legitimately represent workers (Carrier and Kalb 2015). This lack of legitimacy and funding then inhibits unions’ attempts to counter casualization (Pardo and Prato 2012). Linked to both academic and public discourses about the decreasing relevance of unionism has been scepticism of the importance of workplace-based identities and the perception that these identities hold less potential for demanding social justice than those linked to traits like gender, race and sexuality (Carrier and Kalb 2015). While the unions studied in this issue had differing responses to threats to their legitimacy, each attempted to link gendered, raced and intra-familial aspirations to working conditions. Their success (in Mexico) or failures (in Zambia’s North West) in making these linkages salient to workers greatly influenced the outcome of these projects of union maintenance and revival.

Legal and political structures impeded unionization in each of the countries studied. Durrenberger (2007) sees union effectiveness as driven by an environment of stable work, union friendly laws and community acknowledgement of the divergence of interests between workers and management.^1^ Under political economies commonly associated with austerity and those economic and legal structures which IFIs have long argued are necessary for development, such conditions are rarely present. At best, unions are presented as the ‘labour market partners’ of corporations, within an ‘efficient’ capitalist market (Spyridakis 2012 and 2018). However, many states support measures against compulsory unionization and enact legal ‘reforms’ that encourage flexibility, restructuring and innovation (Spyridakis 2013). Across several of the countries explored, unions have either never had or have lost the right to strike and to bargain effectively for wages. Further, left-wing political parties in the Anglo-sphere increasingly downplay their relationships with the union movement (Durrenberger and Erem 2005 and Mollona 2009). Current assaults on the legitimacy of Global North unions mirror the defenestration of unions in the Global South. Unions were crucial to decolonization and democratization in many countries (Cooper 1996 and Larmer 2007). However, development planners, particularly since the 1980s, have encouraged low wages and anti-unionization measures as ways to counter an ‘urban bias’, which they perceived to be slowing national development (famously Lipton 1977, see also Bezemer and Headey 2008). IFI-driven reforms frequently focus on shrinking (unionized) civil services and liberalizing labour markets. While economists have long appreciated the damage done to developing nations by the privatization of public services, the sale of state assets and the reduction of tax-revenue, it has primarily been anthropologists who have described the harm done to the citizenry by the destruction of workers’ rights and salaries (cf Ferguson 1999 and Mollona 2009). While unions have been noted in such studies, they are often only explored through their enfeeblement, with failures to protect workers, confront managers or raise wages attributed to legal disempowerment, technical ineptitude or corruption (Burawoy 1972 and Lee 2017). In detailing unions’ attempts to maintain or restore their relevance, we add nuance to some workers’ depictions of union corruption (see Geenen) and explore other workers’ subversion of their unions (see De la O and Zlolniski).

This special issue builds upon emerging studies exploring unions’ attempts at revival under austerity (Fredricks 2018, Rizzo 2017 and Spyridakis 2017). It notes that many of the tactics used by unions enable co-optation by management, the state or capital, while it also describes how co-dependence between unions and employers is conceptualized (and often encouraged) by workers and the community they are drawn from. Ethnographers including Rizzo (2017) have taken an instrumentalist approach to unionism. They analyse unions through their ability to perform tasks like dispute resolution and to deliver tangible benefits to their members, frequently arguing that delivering these benefits necessitates a fructuous relationship with management. Geenen (this issue) expands upon this framework, exploring how unions’ instrumentality interacts with Congolese norms of patronage and resource redistribution. She observes that in the DRC, low wages incentivize workers to run for union office even if they do not believe in unionist ideologies and she describes how a company finances its favoured candidates’ election campaigns. Yet Geenen claims that employees expect their union representatives to receive cash gifts from management, which union leaders then gift to or share with workers. Unions’ inability to combat corrupt national and international economic elites encouraged new tactics to assist with workers’ livelihoods (receiving and redistributing funds), even while these tactics enable great leverage for management. Linked to the neoliberalism found in all our works is a reduction of the state’s regulatory role, with the companies often regulating themselves. When unions are involved in this process, they are presented as partners or though other discursive devices that hide their weakness and dependence vis-à-vis management. For example, Kapesa and McNamara describe unionists and company HR conceptualizing themselves as the joint ‘household heads’ of the workplace ‘family’, working together to motivate and discipline employees.

Unions’ willingness to work with management can be interpreted through classic Gramscian and Leninist understandings, with the unions’ increasing role in management seen as an expropriation of labour through an expropriation of class consciousness (Kieran 2010). However, while this special issue explores how current models of capitalism appropriate unionists’ labour (see Soul), we also foreground that this appropriation, like resistance or renewal, occurs within community-embedded understandings of personhood, obligation and entitlement. When Geenen argues that the workers she observed did not see the unions as corrupt for accepting payments from management, she links this to Congolese social norms, where receiving financial patronage creates few obligations. In Zambia, many miners found it reasonable that those responsible for ones’ salary and conditions (union leaders and management) take on the obligations and entitlements of ‘household heads’, yet there was substantive disagreement, born from differing kinship expectations, over what obligations being a household head entailed. Much of our work focuses on the unionist identities and structures that are both responding to unions’ disempowerment under neoliberalism and that reconcile transnational union ideologies with local cultural logics and histories.

## Anthropologies of unionism and labour

While trade unions have appeared in classic labour ethnographies, only recently have many works focused on the practices and discourses of union organizing and workplace action. Several significant advances have occurred within this journal. The *Workers of the World* forum series interrogates the links between multiple, locally embedded working classes, their representatives and mobile global capital (Lem and Marcus 2019). Our works are deeply indebted to the insights of Lazar and Sanchez’s (2019) special issue on Labour Politics in an Age of Precarity. Another key event was the International Labour Workshop organized by Paul Durrenberger at the University of Iowa, where an international group of anthropologists and sociologists discussed the relationship between work and trade unions in a period of great depression. This workshop ended in the edition of a volume edited by Paul Durrenberger by the University of Colorado Press in 2017. This book and other union ethnographies inspired by Durrenberger’s network (see Zlolniski 2019) attempt to define unions’ structural traits. An ongoing lacuna in this field is the distinction between shop-floor ethnographies and studies of union offices. Labour anthropology has typically focused on the former, seeing unionists through workers’ (often disappointed) eyes (Burawoy 1972 and Lee 2017). Union ethnographies have analysed the latter, exploring strikes and organizing campaigns and comparing union narratives of epic struggle to the mundanities of union office work (Werbner 2014). In our papers, we attempt to reconcile this disjuncture, exploring union activities in the workplace and linking these to organizing strategies and to the homes and communities that workers create.

Rubbers and Roy (2015) describe the analytic confusion surrounding the label ‘union’. Authors assume that all workers, along with union managements, and even business syndicates share common meanings of unionism, and in doing so overlook how unionism is imaged and organized in different places at different times. In responding to this confusion, we draw from normative definitions provided by union scholars and link them to the way unionism was described by workers, their shop-floor representatives and professional union staff. Durrenberger (2017) and Spyridakis (2017) describe trade unions as organized entities for concerted workers’ action. According to Hyman (2001), unions are often inclusively described as labour market parties or as ‘social partners’. As argued elsewhere (Spyridakis 2017), Hyman (2001) provides an all-inclusive operational definition, which conceptualizes unions as voluntary organizations emerging from civil society’s needs for representativeness, protection, negotiation and advancement of their members’ interests. Such a lose definition enables trade unions to be seen as anything from combatant organizations, to bargaining associations, or even as merely agencies that satisfy ‘economistic’ claims. This loose definition equates unions with any kind of organization seeking to meet its members’ interests, including employers’ associations, and it presumes ‘civil society’ as a peaceful and harmonious assemblage of stakeholders whose existence is based on legally and rationally defined motives. In the European context, trade unions have typically been defined by certain characteristics, which were consolidated after the Second World War: Unions a) are representative organizations speaking for and claiming several social rights on behalf of their members, while preserving a relative autonomy in relation to them; b) are legally recognized by employers and the public opinion, while undertaking several responsibilities; c) are structurally embedded in the institutionalized form of class struggle; d) serve as intermediaries between state and society, politics and economy, labour and capital following ad hoc tactics and policies; e) are massive organizations with specific bureaucratic structures which entails the possible existence of a relative autonomy of the organization from its members (Paleologos 2006: 76).

In contrast to these normative definitions, Lazar and Sanchez’s (2019) issue of *Dialectical Anthropology* explored how the meanings, as much as the practices of unionism, have changed in response increased precariousness. We expand upon both Durrenberger (2017) and Lazar’s (2017) analysis, conceptualizing unions through locally-embedded histories, cultures and collective ethical-political projects. Our approach aims to connect the interpretive tradition of cultural anthropology (with its focus on ethics, meanings and imagination) to the specific political economies of the areas we study. Further, we link unions to the (much more developed) anthropology of labour, arguing that unions influence the worker identities and concepts of the good life that these works increasingly focus upon (as shown in classic ethnographies of work like Moodie and Ndatshe 1994 and Nash 1993). For example, in direct comparison with Bates’ (1972) claim that ethnicity was not allowed to carry over into the field of Zambian labour relations, Kapesa and McNamara argue Zambian narratives of unionism evolved through, and in conjunction with, the same political and economic histories that encourage Zambians to see themselves as members of various tribes. They explore how unions’ association with industrialisation in Central and Eastern Zambia convolves the kinning practices of trade unionism (c.f. Lazar 2017) with the responsibilities of Bemba kinship, but not those of Kaonde men. They argue that what it means to be a ‘unionist’ in Zambia has long drawn from, and shaped, what it means to be Bemba.

Inspired by Lazar (2017 and Keskula and Sanchez 2019), several of our papers use ethnography to explore unions as collective ethical-political projects, while unpacking the norms and cultures from which these projects emerge. For instance, Soul (this issue) scrutinizes the often naturalized concept of ‘union membership’. She argues that in Argentina, ‘membership’ is an active relationship, which creates and affirms the union as a collective ethical being. She then shows how daily practices of membership enable familiarity between differing generations of Argentine steel workers, guiding and challenging Argentine norms of intra-generational interaction. This special issue therefore expands upon the limited literature that conceptualizes unionism as an ethical-political project, and as a kin relationship, by embedding these projects in histories and cultural norms that shape collective action and worker identities. This allows us explore what encourages workers to unionize and how unions remain meaningful in the lives of their members, while also analysing what ‘worker’ and ‘unionist’ mean in times of development and austerity.

A surprisingly important difference between our special issue and much of the trade union anthropology is our focus on unions within the workplace and the links we draw between the anthropologies of unionism and labour (c.f. Rubbers and Roy 2015 and Nash 1993). A substantive section of trade union ethnography describes professional union organizers, often exploring uplifting political campaigns and complex court cases, while leaving shop-floor analysis to those (above) who focus on workers (Ashraf and Prentice 2019, Durrenberger and Erem 2005 and Werbner 2014). Several of our papers explore organizing activities, yet they attempt to link these to day-to-day union work. We detail the union activities that occur in the small, poorly-funded offices where local union staff tend to the daily needs of members and the incidents on the shop-floor that lead to workers describing their unions as corrupt or useless (c.f. Keskula and Sanchez 2019). Our ethnographies explore how these, often banal, activities shape how workers and community members understand unions’ potential for political action and how they form their worker identities. For example, Bithymitris and Spyridakis (this issue) describe how daily interactions between migrant and ethnically Greek workers transformed disparate experiences of exploitation into a unified class consciousness. De la O and Zlonliski (this issue) analyse how the feminization of a traditionally male workforce has enabled female leaders to more effectively link the workplace, union and community.

Our focus on how unions influence the workplace enables us to foreground unions within the anthropology of labour. Labour ethnography has increasingly focused on concerns like kinship, personhood and affect, analysing a worker simultaneously at their place of employment and through how their worker identities respond to and shape their community (Kasmir and Carbonella 2008 and Harvey and Krohn-Hansen 2019). Several of our articles unpack the role of unions in creating workers’ subjectivities in the home and work spaces (cf. Sanchez 2016). For example, Soul (this issue) argues that Argentine unions are crucial for enabling community members’ political action. These insights are part of a broader trend within anthropology to explore how subjectivities enable and create markets, and several papers within our special issue argue are that ‘unionist subjectivities’ guide and shape personhood in communities and workplaces affected by neoliberalism.

## Union relationships, economic anthropology and the anthropology of trade unions

Our special issue has been inspired by recent studies that explore how capitalism is enacted through affective relationships and moral norms. Building upon authors including Bear (2015), Shever (2012) and Ho (2009), we conceptualize capitalist political economies as enacted through relationships and concepts of self, family and community. In this understanding, expectations of kinship, gender and class shape, mediate and mitigate labour markets (Narotzky 2018 and McNamara 2019). We place unions within this anthropological literature, showing how they enable the relationships and moral projects that made someone a ‘worker’. We demonstrate that, in assisting with workers’ daily attempts to maintain various intra-family and community relationships, unions shape industrial relations regimes and political economies (c.f. Shever 2012). Our work utilizes these understandings to analyse unions’ place in an economy driven by global austerity and intra-national promises of development. Conceptualizing capitalism through projects of self-making also enables us to provide new insights into the relationship between unionism and precarious work and into the specificity of unions among civil society actors.

Labour regimes emerge from the interplay of subjectivities within work and home spaces (Prentice 2015 and Hann and Parry 2018). Workplace subjectivities are linked to identities and obligations within the home and community, which unions both shape and help workers fulfil (Sanchez 2016). To understand how these subjectivities shape capitalist projects, consider the concepts of family found in each of Geenen, Kapesa and McNamara and Soul’s papers. McNamara and Kapesa describe how unions offer cash gifts and moral support, enabling workers to be a ‘provider’ even as their wages and conditions deteriorate. In offering this cash, union leaders are simultaneously drawing from and modelling the Bemba patriarch identity that many workers aspire to, shaping home lives and employment conditions. Geenen’s depiction of Congolese patronage presents similar payments from union leaders to workers as an individuated patronage, lacking an obligation to the wider community. These payments (initially provided to unionists by the companies) then operate more like a wage bonus, linking the workers, through the union, to the mine rather than the community. For Soul, like Shever (2012), the union as a family fulfils workers’ physical and emotional needs as the Argentine state recedes. Yet it also creates them as political subjects, shaping the contours of the capitalist system they will consent to.

Understanding capitalism through affective relationships and moral norms can offer new insights if applied to other key issues in the anthropologies of labour and unionism. A key area where an understanding of the interplay between unionism and labour identities could contribute to the state of the art is the growing body of literature on precarious workers. In Standing’s (2011) influential conceptualisation, the precariat (those who engage in precarious employment) do not understand themselves as workers. However, subsequent studies have rejected this claim, exploring how informal work influences the worker identities through which the precariat both make moral claims and shape local workplace conditions (Spyridakis 2013 and Hann and Parry 2018). For example, precarious workers in Trinidad encourage their own worsening conditions, believing that the alternative is a loss of employment (Prentice 2018). Yet ‘precarious worker’ is both a collection of identities and a political category. It is a term, at least in part, shaped through the politics of trade unions. Kasmir (2018:10) argues that a key goal of analysing precarity is to ‘determine how distinctions among workers are made and unmade’. Studying how unions create and restrict the social category ‘worker’ and the obligations and entitlements this entails is core to this task. Future works could explore how unions encourage individuals to see themselves as ‘precarious workers’, how they influence the meaning of this identity and how this links local capitalisms to global trends.

Similarly, labour struggles are increasingly conceptualized as encompassed within ‘new social movements’. In this context, the meaning of ‘social movement unionism’ has shifted from Von Holdt’s (2003) understanding of a ‘militant, mobile unionism’ (associated with decolonising struggles) to ‘a widespread mobilization of civil society associations that supersedes the notion of class identity’ (Werbner 2014). However, exploring how unionism is enacted at the workplace foregrounds that unions are structurally different to the semi-formal collections of passionate individuals that conceptualisations of civil society often focus upon (see Habib 2005). We therefore join several anthropologies in attempting to understand what makes unions different to other civil society bodies (Rubber and Roy 2015) and through this more accurately detail the potential and pitfalls of union alliances with CSOs and NGOs.

McNamara and Kapesa and De la O and Zlolniski diverge significantly in their analysis of community unionism. For De la O and Zlolniski, engagement with a broader civil society is a wise response to the declining power of (corrupt) union officials, linking workers’ class identities to their concurrently held personhoods, generated through regional history, ethnicity and cultural background. However, on Zambia’s Copperbelt, Kapesa and McNamara argue that the union’s long entwinement with daily life ensures that unionist identities and class interests reflect the concerns of everyday Copperbelt residents. Calls for financial cooperation between unions and CSOs in Zambia’s North West (far from the Copperbelt) signify North Westerners’ lack of faith in the unions to similarly incorporate their identities and concerns, with transfers of cash to civil society groups second-best option that with impedes political mobilization. This disagreement reflects how unionism’s meaning is embedded within local political histories and economies, while it shapes, challenges and often stabilizes class relations. In this special issue, we add to studies of unions political action by linking the shop floor to the community, noting that despite their allegedly diminishing influence, unions are still meaningful in the lives of many people and are often the only formal social association they join (Lazar 2017). If unions are to contribute to social change, it is important to understand how the meaning of unions’ official reports and national negotiations are understood by workers and shop stewards, as well as how (and if) these reports and negotiations reflect their aspirations as workers.

Bithymitris and Spyridakis’ *Union Radicalism* versus *the Nationalist Upsurge* describes how the recent global economic crisis and the concomitant austerity policy exacerbated challenges to organized labour, both in terms of inequality in resource allocation and in the rise of reactionary politics. Using ethnography in the Greek shipbuilding and ship repairing industry, Bithymitris and Spyridakis detail how radical political unionism responded to employer hostility and escalating far right violence. Their paper tells the story of the Trade Union of Metal Workers of Attica and the Shipbuilding Industry of Greece’s battle against social injustice and right-wing extremism and this conflict’s wider importance for labour’s responses to challenges to its legitimacy.

Julia Soul’s *The Union is like a Family Father, the Chief* investigates the relationship between the Argentinean Metalworkers Union (UOM) and its workers. It conceptualizes union membership as a relational experience, crossed not only by administrative or bureaucratic bonds, but also by political, ideological and cultural ones. Through comparing the membership experiences of two generations of Argentinian steelworkers—the generation that experienced the restructuring process and the generation that accessed a ‘global’ workplace—the article assesses the daily practices that reproduce membership and shape workers’ as political subjects, who respond to Argentinean austerity.

Similarly focusing on the affective relationships embedded within unions, Kapesa and McNamara’s *We Are Not Just a Union, We Are a Family* argues that Zambian understandings of unionism developed through similar political economic processes to those that generated ‘tribes’. McNamara and Kapesa claim that economic structures that enable concepts of the good life more commonly found among Bemba speakers have been naturalized into Zambia’s mining unions, guiding union policy and practice in a manner which limits North Western Zambians’ union participation. Utilizing Lazar (2017) understanding of unionism as kinship, they explore how Zambians of various tribes attempt to utilize unions to achieve what they see as human flourishing and social justice and demonstrate that intra-national unionisms are co-created through (and influence) local cultural norms and political histories. This leads them to be sceptical of the way alliances with Civil Society Organizations are frequently presented as a panacea for unionism’s struggles.

Geenen’s *Patronage, Social Proximity and Instrumentality in the Mining Industry in the Democratic Republic of Congo* examines workers’ votes during union elections. She argues that workers voted for candidates who were perceived to be patrons, a status acquired by improving labour conditions and securing jobs. As such, workers voted following an evaluative rationale and used voting to shape their working conditions. However, Geenen also notes that the grip of the management upon the chosen union delegation remained very tight, both during and after the elections.

Finally, De la O and Zlolniski’s *At the Crossroads* examines the push for independent labour unions in export-oriented industries in the Mexico-USA border. Using a critical political economy perspective, they contend that the production regimes of these twin export industries along with the weakening of the state and traditional patronage-based business unions have created the conditions for the re-emergence of independent labour unions which workers mobilize for labour demands as well as human and citizenship rights. Analysing two labour strikes De la O and Zlolniski describe model of social movement unionism that has been making inroads in Mexico and other Latin American countries as workers confront the challenges of advanced labour regimes in the twenty-first century.

## Footnotes

1 His five conditions are as follows: 1) workers’ notions of their own interests separated from those of the management; 2) certain promotion of self-organization in the productive terrain; 3) the possibility of mutual discussion of concerns; 4) the existence of a ‘union friendly’ political and legal environment; 5) unions as being able to show members how to address work issues and concerns (Durrenberger: 2007: 74).
